# Diagnostic classification of cancers using DNA methylation of paracancerous tissues

**DOI:** 10.1038/s41598-022-14786-7

**Published:** 2022-06-23

**Authors:** Baoshan Ma, Bingjie Chai, Heng Dong, Jishuang Qi, Pengcheng Wang, Tong Xiong, Yi Gong, Di Li, Shuxin Liu, Fengju Song

**Affiliations:** 1 School of Information Science and Technology, Dalian Maritime University, Dalian, 116026 China; 2 Department of Mechanical Engineering, University of Houston, Houston, TX 77204 USA; 3 Department of Neuro Intervention, Dalian Medical University Affiliated Dalian Municipal Central Hospital, Dalian, 116033 China; 4 Department of Nephrology, Dalian Medical University Affiliated Dalian Municipal Central Hospital, Dalian, 116033 China; 5 Department of Epidemiology and Biostatistics, Key Laboratory of Molecular Cancer Epidemiology, Tianjin, National Clinical Research Center of Cancer, Tianjin Medical University Cancer Institute and Hospital, Tianjin, 300060 China

**Keywords:** Cancer, Computational biology and bioinformatics, Biomarkers

## Abstract

The potential role of DNA methylation from paracancerous tissues in cancer diagnosis has not been explored until now. In this study, we built classification models using well-known machine learning models based on DNA methylation profiles of paracancerous tissues. We evaluated our methods on nine cancer datasets collected from The Cancer Genome Atlas (TCGA) and utilized fivefold cross-validation to assess the performance of models. Additionally, we performed gene ontology (GO) enrichment analysis on the basis of the significant CpG sites selected by feature importance scores of XGBoost model, aiming to identify biological pathways involved in cancer progression. We also exploited the XGBoost algorithm to classify cancer types using DNA methylation profiles of paracancerous tissues in external validation datasets. Comparative experiments suggested that XGBoost achieved better predictive performance than the other four machine learning methods in predicting cancer stage. GO enrichment analysis revealed key pathways involved, highlighting the importance of paracancerous tissues in cancer progression. Furthermore, XGBoost model can accurately classify nine different cancers from TCGA, and the feature sets selected by XGBoost can also effectively predict seven cancer types on independent GEO datasets. This study provided new insights into cancer diagnosis from an epigenetic perspective and may facilitate the development of personalized diagnosis and treatment strategies.

## Introduction

Cancer continues to be a leading cause of mortality worldwide^1^. On the basis of the GLOBOCAN 2020 estimates of cancer incidence and mortality reported by the International Agency for Research on Cancer, an estimated 19.3 million new cancer cases (18.1 million excluding nonmelanoma skin cancer) and almost 10.0 million cancer deaths (9.9 million excluding nonmelanoma skin cancer) occurred in 2020^2^. Prediction of cancer stage and type as the important applications in cancer diagnosis are crucial for planning appropriate treatments.

Previous studies related to cancer diagnosis mainly focused on molecular data of tumor tissues^3–6^. Broët et al. proposed a new statistic for identifying gene expression features that detected tumor progression^4^. Rahimi et al. developed the highly time-efficient benders decomposition algorithm for the forest formulation (BDForest) to solve the problem of finding the similarity between different cancers, which is beneficial in classifying the stage of tumors^7^. Some studies have investigated molecular data of human pan-cancer and identified key biomarkers for prognosis and diagnosis of pan-cancer^8–10^.

Paracancer is the place where tumor growth and metastasis start. Wang et al. reported that the complement and angiogenesis pathways correlated with cancer progression were activated in the paracancerous tissues^11^. This finding revealed that the changes in paracancerous tissues are crucial complements to the conventional analysis of tumor tissues. Clinically, paracancerous tissues are more accessible to obtain than tumor tissues. Therefore, cancer stage and type prediction based on molecular data of paracancerous tissues may provide valuable information on understanding tumor stage progression and contribute to developing new approaches for cancer diagnosis. To the best of our knowledge, there have been no systematic studies that utilize data of paracancerous tissues to classify cancer stage and type.

In this study, we utilized extreme gradient boosting (XGBoost)^12^ to discriminate tumor stage based on DNA methylation profiles of paracancerous tissues. The proposed XGBoost approach obtained better predictive performance than the other four machine learning methods. Moreover, our model extracted significant features from genome-wide DNA methylation profiles. GO enrichment analysis provided evidence that DNA methylation biomarkers of paracancerous tissues were closely associated with the progression of tumor stage. Additionally, we employed the XGBoost algorithm to build a multiclass classifier, which can accurately identify nine different cancers on the basis of DNA methylation profiles of paracancerous tissues from TCGA. The feature sets selected by the XGBoost model have high accuracy in cancer type prediction on independent GEO datasets.

## Results

### Predictive performance comparison for cancer stage prediction

**Figure 1:**
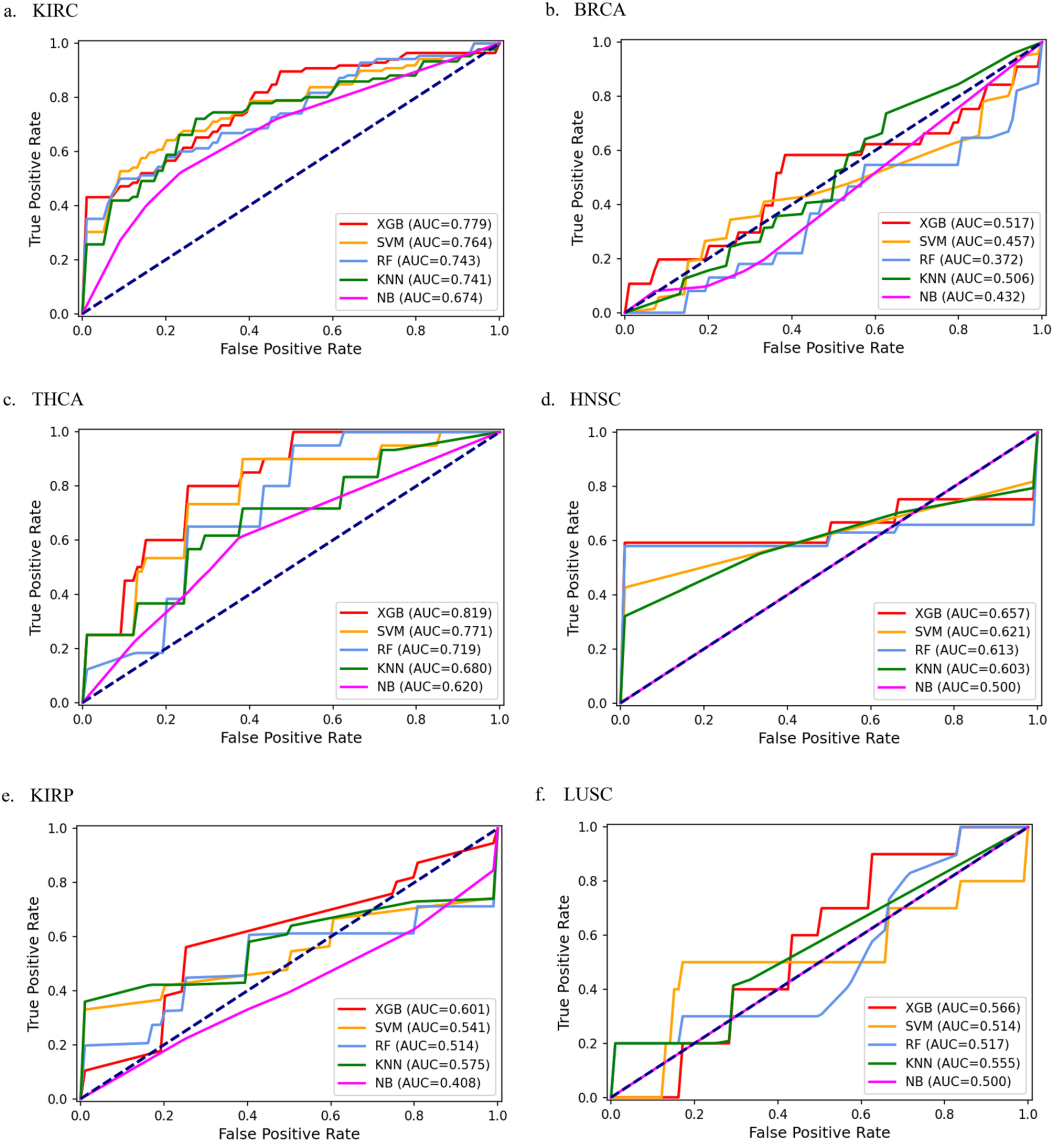

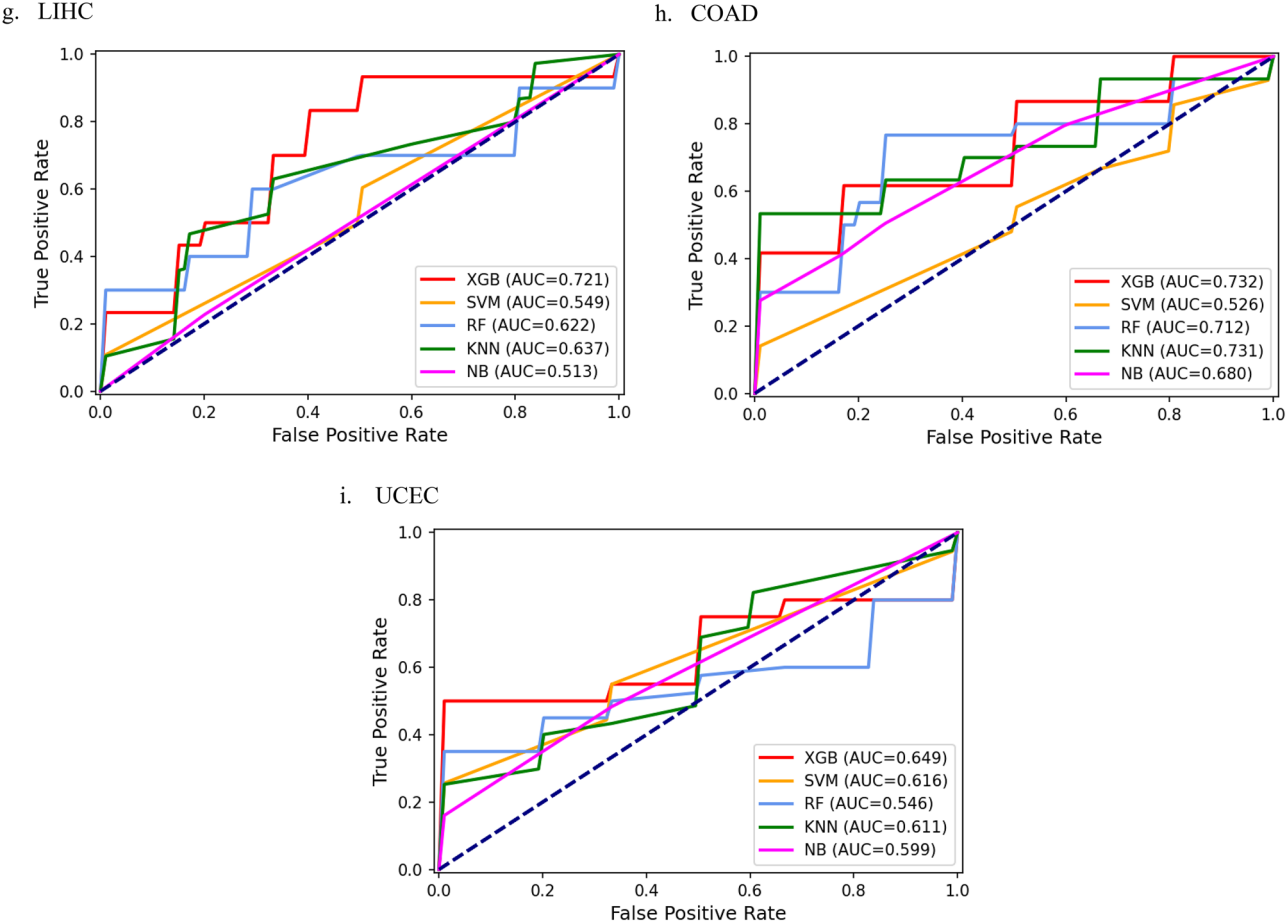
The ROC curves of XGBoost, SVM, RF, KNN and NB on nine datasets. (**a**) KIRC, (**b**) BRCA, (**c**)THCA, (**d**) HNSC, (**e**) KIRP, (**f**) LUSC, (**g**) LIHC, (**h**) COAD, (**i**) UCEC.

For cancer stage prediction, we evaluated the predictive accuracy of five classification methods by fivefold-cv on nine datasets in this study. The area under the curve (AUC) of the receiver operating characteristic curve (ROC), the area under the precision-recall curve (AUPR), accuracy (ACC), matthews correlation coefficient (MCC), Precision and Recall for different models on nine datasets were calculated as shown in Table 1. The AUC, AUPR, ACC, MCC, Precision, Recall of these algorithms obtained in each fold of all datasets can be found in Supplementary Table S1–S6, respectively. To compare the XGBoost model more intuitively with other machine learning methods, we further plotted ROC curves of five classification models, which were shown in Fig. 1.

**Table 1:** Comparison of prediction performance of different classification models on different datasets.

Cancer type	Model	AUC	ACC	AUPR	MCC	Precision	Recall
KIRC	XGBoost	**0.780**	**0.675**	**0.842**	0.353	0.703	0.747
	SVM	0.764	0.650	0.827	0.298	0.700	**0.750**
	RF	0.743	0.600	0.817	0.205	0.643	0.683
	KNN	0.741	0.669	0.795	**0.392**	**0.807**	0.542
	NB	0.674	0.656	0.795	0.350	0.747	0.631
BRCA	XGBoost	**0.516**	**0.789**	**0.323**	**0.079**	**0.200**	0.040
	SVM	0.456	0.779	0.292	0.000	0.000	0.000
	RF	0.372	0.779	0.184	0.000	0.000	0.000
	KNN	0.505	0.726	0.263	-0.051	0.050	0.050
	NB	0.432	0.632	0.207	-0.126	0.133	0.080
THCA	XGBoost	**0.819**	**0.735**	0.538	0.137	**0.400**	0.183
	SVM	0.773	**0.735**	0.566	0.202	**0.400**	0.250
	RF	0.719	0.733	0.441	0.045	0.200	0.050
	KNN	0.681	0.662	**0.604**	0.103	0.333	0.317
	NB	0.620	0.679	0.515	**0.259**	**0.400**	**0.550**
HNSC	XGBoost	**0.658**	**0.840**	0.925	0.000	**0.840**	**1.000**
	SVM	0.622	0.840	**0.928**	0.000	0.840	1.000
	RF	0.614	0.820	0.924	-0.022	0.838	0.978
	KNN	0.603	0.840	0.917	0.000	0.840	1.000
	NB	0.500	0.840	0.920	0.000	0.840	1.000
KIRP	XGBoost	**0.600**	0.444	0.683	0.087	0.406	0.670
	SVM	0.541	0.444	0.680	**0.110**	0.404	**0.760**
	RF	0.514	**0.511**	0.621	0.011	0.533	0.549
	KNN	0.576	0.467	**0.709**	0.089	**0.632**	0.402
	NB	0.407	0.422	0.582	-0.163	0.450	0.404
LUSC	XGBoost	**0.565**	**0.828**	0.180	0.000	0.000	0.000
	SVM	0.513	0.828	0.197	0.000	0.000	0.000
	RF	0.517	0.828	0.315	0.000	0.000	0.000
	KNN	0.556	0.828	0.282	**0.087**	**0.200**	**0.100**
	NB	0.500	0.828	**0.586**	0.000	0.000	0.000
LIHC	XGBoost	**0.721**	0.675	0.477	-0.087	0.000	0.000
	SVM	0.550	**0.725**	**0.645**	0.000	0.000	0.000
	RF	0.625	0.725	0.483	0.000	0.000	0.000
	KNN	0.638	0.725	0.473	**0.087**	**0.200**	**0.100**
	NB	0.513	0.725	0.608	0.030	0.100	0.067
COAD	XGBoost	**0.735**	0.589	0.671	0.291	0.617	0.550
	SVM	0.527	0.618	0.658	0.000	0.000	0.000
	RF	0.713	0.643	0.599	0.340	0.433	**0.567**
	KNN	0.732	0.582	**0.737**	0.188	0.333	0.433
	NB	0.682	**0.693**	0.689	**0.374**	**0.633**	**0.567**
UCEC	XGBoost	**0.650**	**0.667**	0.688	**0.296**	0.300	**0.400**
	SVM	0.617	0.638	**0.699**	0.000	0.000	0.000
	RF	0.547	0.581	0.608	0.061	0.067	0.200
	KNN	0.612	0.557	0.562	0.124	0.333	0.350
	NB	0.600	0.614	0.605	0.217	**0.567**	0.400

*KIRC* kidney renal clear cell carcinoma, *BRCA* breast invasive carcinoma, *THCA* thyroid carcinoma, *HNSC* head and neck squamous cell carcinoma, *KIRP* kidney renal papillary cell carcinoma, *LUSC* lung squamous cell carcinoma, *LIHC* liver hepatocellular carcinoma, *COAD* colon adenocarcinoma, *UCEC* uterine corpus endometrial carcinoma, *XGBoost* Extreme gradient boosting, *SVM* Support vector machine, *RF* Random forest, *KNN* K-Nearest Neighbor, *NB* Naive Bayes. *AUC* the area under the receiver operating characteristic curve, *ACC* accuracy, *AUPR* the area under precision-recall curve, *MCC* matthews correlation coefficient. Significant values are in bold.

We compare the AUC values of the five classification algorithms on nine datasets. We observe that XGBoost obtains significantly better results than baseline algorithm NB on all nine datasets, and it improves the average AUC by 22.6%. SVM outperforms NB on eight out of nine datasets and it improves the average AUC by 8.8%. RF performs better than NB on seven out of nine datasets and it improves the average AUC by 8.8%. KNN is able to outperform NB on all nine datasets and it improves the average AUC by 14.4%. We notice that the performance of NB is superior to the baseline (i.e. AUC = 0.5) on five out of nine datasets, further indicating that the other four classification models yield higher performance.

The results suggest that XGBoost is far better than other classification models. The average AUC score of XGBoost is 0.672, which is 12.8%, 22.6%, 7.2% and 12.8% higher than SVM, NB, KNN and RF, respectively. Moreover, XGBoost substantially improves the predictive performance of stage prediction for cancer patients on nine datasets. Compared to the model with the worst prediction result, XGBoost increases the AUC values 15.7%, 38.7%, 32.1%, 31.6%, 47.4%, 13%, 40.5%, 39.5% and 18.8%, respectively.

In addition, we calculate the ACC values of the five different classification models on nine datasets. Our results show that XGBoost predominantly outperforms NB, KNN, RF, and maintains comparable performance compared to SVM. XGBoost is superior to other models on six out of nine datasets and its average ACC is 0.694 that is 2.5%, 3.1% and 0.4% higher than NB, KNN and RF, respectively.

Furthermore, XGBoost is still more competitive than other methods in terms of AUPR. The average MCC of XGBoost is 0.128, which is 88.2%, 80.3%, 13.3% and 21.9% higher than SVM, RF, KNN and NB, respectively. The average Precision of XGBoost is 0.385, which is 48.1% and 27.5% higher than SVM and RF, respectively. The average Recall of XGBoost is 0.399, which is 30%, 18.8% and 9% higher than SVM, RF and KNN, respectively.

In summary, these results clearly indicate that XGBoost achieves better performance on nine datasets by assessing AUC. Moreover, XGBoost marks the best results in most evaluation metrics, suggesting that it is superior to other classification methods. Specifically, the maximum AUC scores for nine datasets are 0.780 (KIRC), 0.516 (BRCA), 0.819 (THCA), 0.658(HNSC), 0.600(KIRP), 0.565(LUSC), 0.721(LIHC), 0.735(COAD) and 0.650(UCEC), respectively.

### Gene ontology enrichment analysis

**Figure 2:**
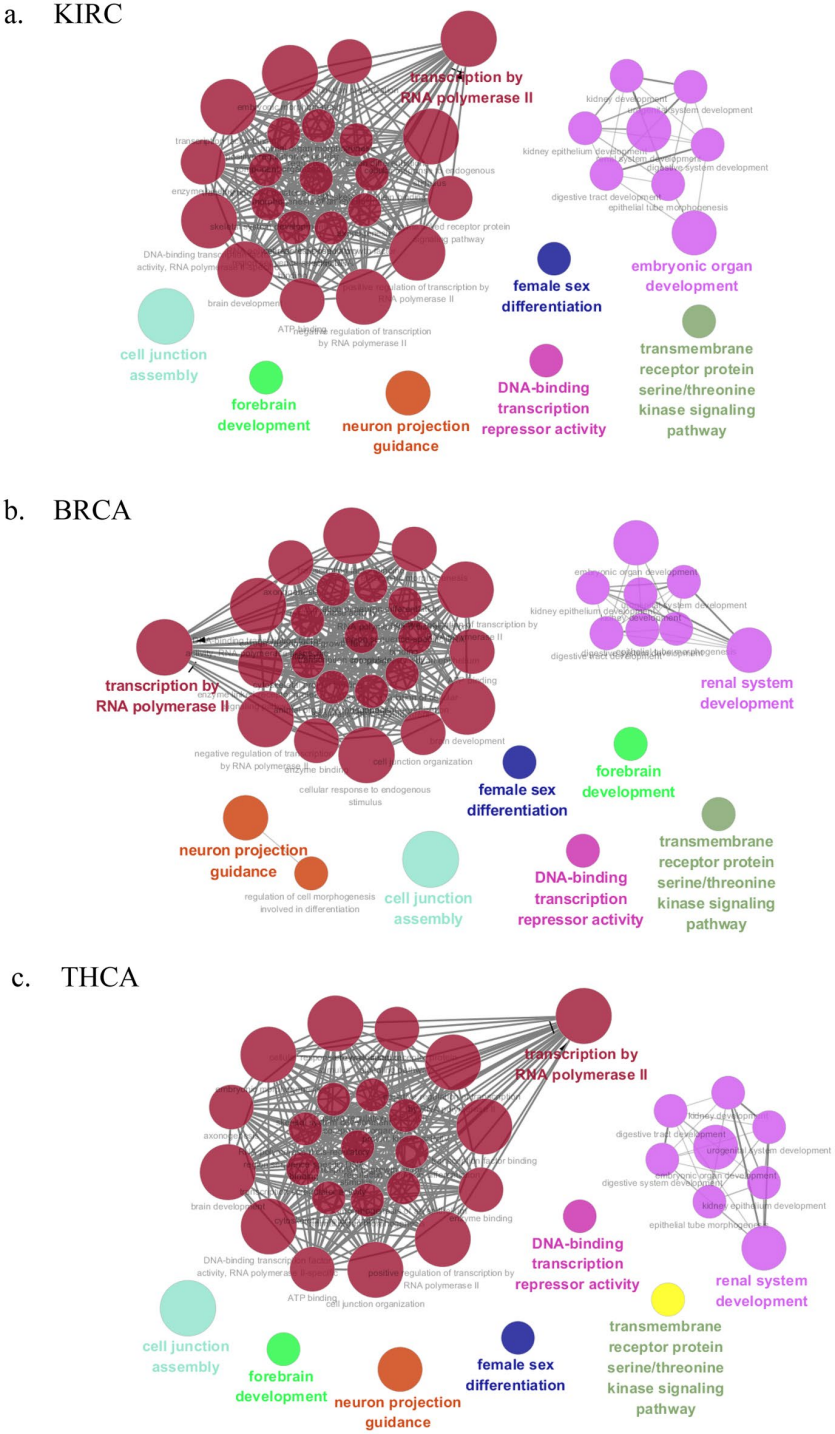

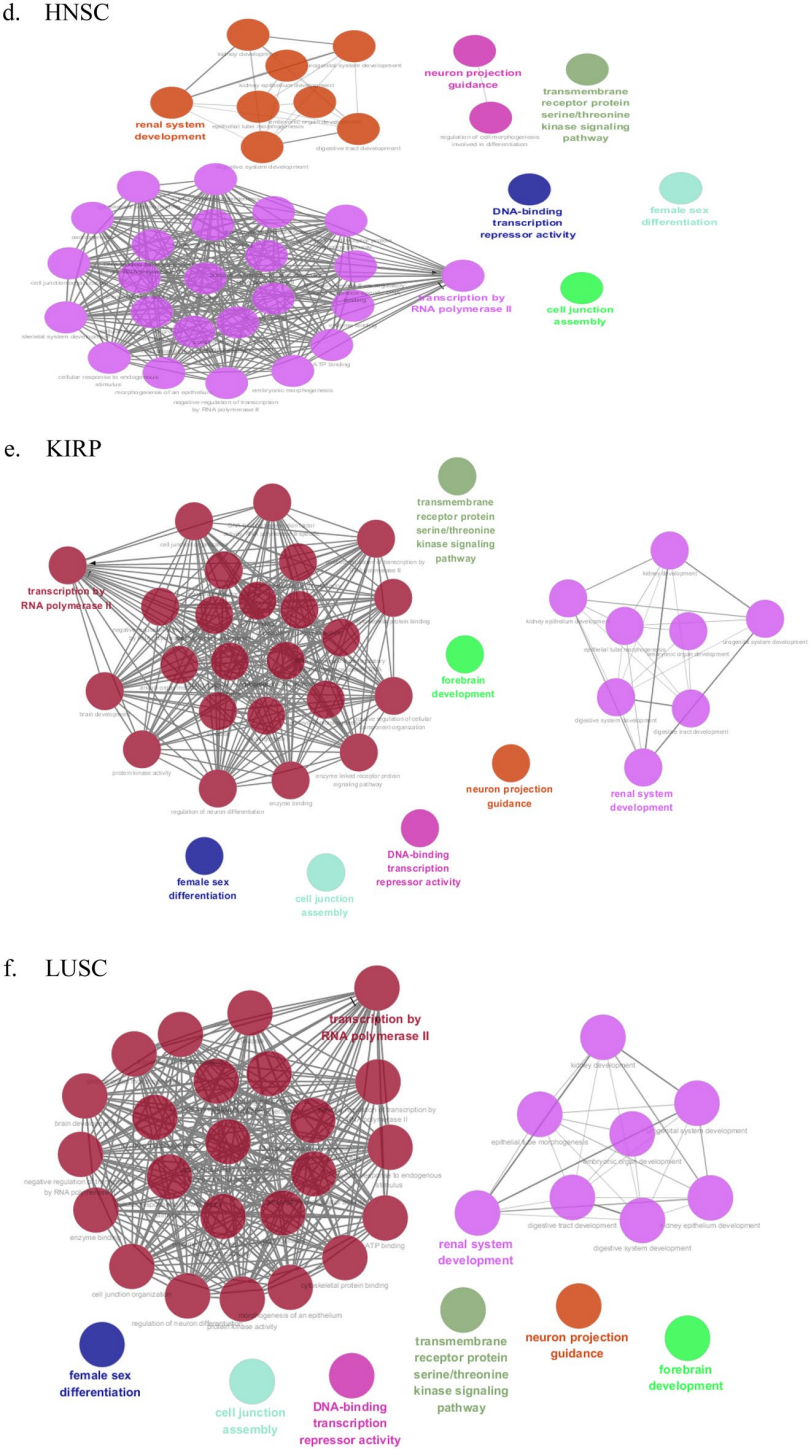

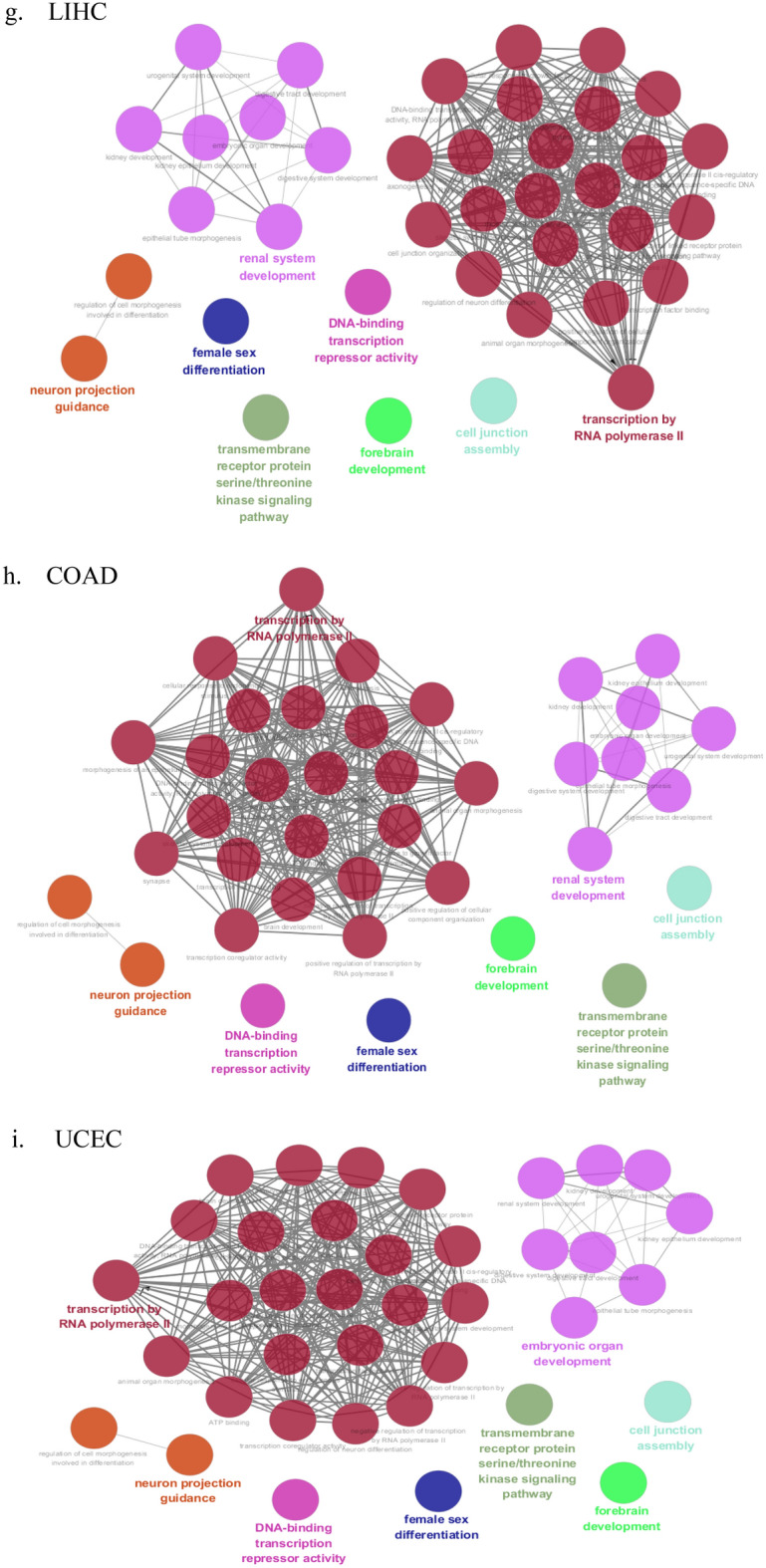
Cluego analysis for GO terminology on nine datasets. Node: GO term; the bigger the node, the smaller the *P* value; Each line indicates the correlation between functions, and a larger kappa coefficient represents the line is more thicker; different colors denote the function enrichment classification of GO terms. Networks were generated with ClueGO (version 2.5.7) in Cytoscape (version 3.6.0) (http://apps.cytoscape.org/apps/cluego). (**a**) KIRC, (**b**) BRCA, (**c**) THCA, (**d**) HNSC, (**e**) KIRP, (**f**) LUSC, (**g**) LIHC, (**h**) COAD, (**i**) UCEC.

The GO analysis can identify biological pathways for revealing the relation between tumor progression and the CpG sites derived from the XGBoost model using the DNA methylation data of paracancerous tissues. GO analysis was conducted with the missMethyl package based on the top 10% significant CpG sites of nine cancers. Then we respectively obtained 147, 154, 155, 153, 151, 150, 152, 150, 152 important GO terms for KIRC, BRCA, THCA, HNSC, KIRP, LUSC, LIHC, COAD and UCEC. All GO enrichment results of nine datasets can be found in Supplementary Table S7–S15, respectively. From GO analysis, many enriched GO terms for each cancer were determined. However, it was difficult to analyze them one by one, and these GO terms may be redundant. Therefore, we elected to cluster them into more representative terms using the Cytoscape plugin ClueGO. ClueGO network diagram was visualized based on the following basic parameters: kappa coefficient was set to 0.1, three categories of GO were used for ontology files, where each node and line represented a term and the correlation between the terms, respectively. Different node colors denoted the classification of terms according to the functions. ClueGO network diagrams of nine cancers were depicted in Fig. 2. The results from ClueGO enrichment clearly illustrated that the most significant GO terms were transcription by RNA polymerase II, transmembrane receptor protein serine/threonine kinase signaling pathway, neuron projection guidance, female sex differentiation, DNA-binding transcription repressor activity, forebrain development and cell junction assembly in KIRC, BRCA, THCA, HNSC, KIRP, LUSC, LIHC, COAD and UCEC. Among the most significant GO terms, we determined common GO terms related to tumor progression in nine cancers. Further analyses for these GO terms can be found in the discussion.

### Classification of distinct cancers using XGBoost

Based on DNA methylation data of paracancerous tissues, we exploited XGBoost to construct a tumor specific classifier for cancer type prediction. Table 2 presented all prediction results for TCGA and GEO datasets, respectively. XGBoost model obtained an accuracy of 100% for KIRC, 100% for BRCA, 100% for THCA, 100% for HNSC, 100% for KIRP, 100% for LUSC, 100% for LIHC, 100% for COAD and 100% for UCEC, exhibiting an average accuracy of 100% across all nine cancer types. Thirty-one features ranked by their importance scores were selected by XGBoost and validated using independent GEO datasets. For GEO datasets, XGBoost achieved an accuracy of 100% for KIRC, 97.6% for BRCA, 97.6% for THCA, 96.6% for HNSC, 71.4% for LUSC, 65.1% for LIHC and 81.8% for COAD, showing an average accuracy of 86.1% across seven cancer types. These results indicated that XGBoost can accurately distinguish different cancer types using DNA methylation data of paracancerous tissues.

**Table 2:** Classification accuracy of XGBoost on TCGA and GEO datasets.

Cancer type	XGBoost (TCGA dataset) (%)	XGBoost (GEO dataset)
KIRC	100	100
BRCA	100	97.6
THCA	100	97.6
HNSC	100	96.6
KIRP	100	–
LUSC	100	71.4
LIHC	100	65.1
COAD	100	81.8
UCEC	100	–
Overall accuracy	100	86.1

*KIRC* kidney renal clear cell carcinoma, *BRCA* breast invasive carcinoma, *THCA* thyroid carcinoma, *HNSC* head and neck squamous cell carcinoma, *KIRP* kidney renal papillary cell carcinoma, *LUSC* lung squamous cell carcinoma, *LIHC* liver hepatocellular carcinoma, *COAD* colon adenocarcinoma, *UCEC* uterine corpus endometrial carcinoma. *XGBoost* Extreme gradient boosting.

## Discussion

In previous studies, researchers mostly paid attention to the tumor itself and sought for the initiation and progression indicators of cancer from tumor tissues. However, more and more studies have suggested that inflammatory microenvironment is closely correlated with tumorigenesis and development. When the tumor develops to a certain stage, the paracancerous tissues are in the state of ischemia and hypoxia, and the increase of autophagy level will also promote the chemotherapy resistance, recurrence and metastasis of cancer, suggesting a poor prognosis^13^. In this sense, certain molecular responses and activities in paracancerous tissues may be related to the characteristics and status of cancer, thus providing potentially useful information for cancer type and stage prediction.

It is well recognized that reliable tumor stage prediction is critical for determining therapeutic strategies. Moreover, tumor treatment is highly dependent on the correct identification of the tumor origins. Tumor growth and metastasis affect paracancerous tissues, thereby it is valuable to investigate the relationship between molecular data of paracancerous tissues and pathological tumor stage.

To explore the potential role of DNA methylation profiles from paracancerous tissues in predicting cancer stages, we exploited the XGBoost algorithm to construct a classification model for cancer progression. We evaluated the performance of five classification methods by fivefold-cv.The comparison results of five advanced machine learning methods on nine datasets showed that XGBoost outperformed other classification models by assessing AUC and performed the best in the majority of metrics.

Previous studies almost utilized DNA methylation of tumor tissues for cancer stage prediction. Ma et al. employed the XGBoost model on the basis of multi-omics data to distinguish early and late stage tumors^14^. For KIRC, it achieved ACC of 0.719 and AUC of 0.797 based on DNA methylation data, whereas our results indicated that the ACC and AUC scores of XGBoost were 0.675 and 0.780, respectively. Deng et al. utilized gene expression and DNA methylation to build three networks for identifying the KIRC stages^15^. The prediction accuracy of the network using DNA methylation profiles was 0.696, which was 3.1% higher than the accuracy of our model. Bhalla et al. identified key biomarkers using gene expression data for distinguishing stages of KIRC^16^. The experiments showed that the model obtained accuracy of 0.726 and AUC of 0.81, both of which were higher than the results of our model. Although our results are lower than the previous results, our study suggests that DNA methylation profiles of paracancerous tissues could possibly be used to identify cancer stages, which may be an alternative strategy for diagnosis and personalized target therapies in the case where tumor tissues are difficult to obtain.

We performed GO analysis for the CpG sites identified by XGBoost model. The results indicate that the enriched GO terms associated with tumor progression are neuron projection guidance, cell junction assembly, transmembrane receptor protein serine/threonine kinase signaling pathway, transcription by RNA polymerase II and DNA-binding transcription repressor activity. Neuron projection guidance and cell junction assembly mainly refer to cellular processes. It is consistent with the knowledge that the defective functioning of cell biological processes is considered to be associated with tumor progression^17^. As a signal transduction pathway contributing to the pathogenesis of cancer, transmembrane receptor protein serine/threonine kinase signaling pathway describes a series of molecular signals initiated by the binding of an extracellular ligand to a receptor on the surface of the target cell^18^. Another GO term, transcription by RNA polymerase II, as the endpoint of signal transduction pathways, is the basis of development and differentiation^19^. The pathogenic mechanisms leading to cancer frequently involve altered signal transduction pathways. Furthermore, there remains a specific GO term of molecular function (MF), DNA-binding transcription repressor activity, which represses or decreases the transcription of specific gene sets. Aberrant regulation patterns at transcriptional level is regarded as a cause of human diseases^20^. Overall, these enriched GO terms demonstrated that underlying regulatory processes may participate in tumorigenesis. Moreover, the biological interpretation of the enriched GO terms suggested the relation between tumor progression and the significant CpG sites, which may serve as surrogate biomarkers for cancer diagnosis.

To our knowledge, it is the first research to establish a classification model for separating tumor stages on the basis of DNA methylation data of paracancerous tissues. Our study gave a systematic assessment of the performances of several machine learning algorithms for discriminating tumor stage and revealed the significance of paracancerous tissues for cancer progression. We also used XGBoost to construct the tumor specific multiclass classifier which can predict cancer type with high accuracy based on DNA methylation of paracancerous tissues. Furthermore, the utility of our model was emphasized by identification of the key CpG sites and GO terms associated with oncogenesis and tumor progression. Altogether, the investigation of DNA methylation profiles from paracancerous tissues may be helpful for understanding cancer progression and discovering new biomarkers. Our findings suggested that paracancerous tissues could be used as surrogate tissues for cancer stage prediction when tumor tissues were quite challenging to obtain.

Nevertheless, our study still has some limitations. First, the main limitation is the small sample size of nine cancer types used in the study. The inherent problem of the small sample size resulted in imprecision of prediction models. Second, we observed that the patients at different stages of KIRC and THCA can be well distinguished, whereas different stages of BRCA patients can’t be clearly separated. The reasons for this are probably tumor heterogeneity and differences in tumor type. Because of tumor heterogeneity, there are obvious individual differences among BRCA patients^21^. Due to the imbalanced sample ratio of early stage and late stage being about 5:1, our model can not achieve better performance on LUSC. In addition, the reason for the low AUC of KIRP on our model may be due to a small sample size. Third, the races of patients include Asian, Black or African-American, White and not available, and White group accounts for the majority. Considering racial differences in cancers, our findings may not be suitable for paracancerous tissue data collected from other races.

In conclusion, our study suggested the potential role of paracancerous tissues in cancer diagnosis. One of our future efforts is to examine the possibility of other molecular data of paracancerous tissues in predicting the stage of tumors. The further application of our findings will contribute to understanding tumor progression and ultimately improving tumor treatment.

## Methods

### Data collection and pre-processing

We obtained DNA methylation profiles (HumanMethylation450; Level 3) and the corresponding clinical data of several cancers from The Cancer Genome Atlas (TCGA)database. Only paracancerous tissue samples were taken into account in the study. Paracancerous tissues in TCGA were represented as normal samples in some previous studies^22,23^. We utilized nine cancer types with the sample size larger than 20, including 160 kidney renal clear cell carcinoma (KIRC) patients, 96 breast invasive carcinoma (BRCA) patients, 56 thyroid carcinoma (THCA) patients, 50 head and neck squamous cell carcinoma (HNSC) patients, 50 liver hepatocellular carcinoma (LIHC) patients, 46 uterine corpus endometrial carcinoma (UCEC) patients, 45 kidney renal papillary cell carcinoma (KIRP) patients, 42 lung squamous cell carcinoma (LUSC) patients and 38 colon adenocarcinoma (COAD) patients. For DNA methylation, we excluded the CpG sites with missing values in more than 20% of samples, and then imputed the remaining missing values using “na.roughfix” function in the “randomForest” package^24^. Table 3 presents the number of early stage samples, late stage samples and DNA methylation profiles for nine datasets.

**Table 3:** The description of TCGA datasets used in this study.

Cancer type	Patient class	Total of patients	Total of methylation profiles
KIRC	Early	71	395,708
	Late	89	
BRCA	Early	74	395,479
	Late	21	
THCA	Early	41	395,661
	Late	15	
HNSC	Early	8	395,363
	Late	42	
KIRP	Early	22	395,392
	Late	23	
LUSC	Early	34	395,680
	Late	7	
LIHC	Early	29	395,564
	Late	11	
COAD	Early	23	395,552
	Late	15	
UCEC	Early	22	395,616
	Late	12	

*KIRC* kidney renal clear cell carcinoma, *BRCA* breast invasive carcinoma, *THCA* thyroid carcinoma, *HNSC* head and neck squamous cell carcinoma, *KIRP* kidney renal papillary cell carcinoma, *LUSC* lung squamous cell carcinoma, *LIHC* liver hepatocellular carcinoma, *COAD* colon adenocarcinoma, *UCEC* uterine corpus endometrial carcinoma.

We also collected clear cell renal cell carcinoma (GSE61441^25^), breast carcinoma (GSE69914^26^), thyroid carcinoma (GSE86961^27^), head and neck squamous cell carcinoma (GSE75537^28^), lung squamous cell carcinoma (GSE94785^29^), liver hepatocellular carcinoma (GSE54503^30^) and colon adenocarcinoma(GSE42752^31^) from Gene Expression Omnibus (GEO). Each dataset contained paracancerous tissues and tumor tissues. The GEO datasets were only utilized for cancer type prediction due to the lack of pathological stage information. Therefore, we selected all paracancerous tissues of the seven datasets for independent validation. Table 4 shows the number of patients and DNA methylation profiles for the GEO datasets.

**Table 4:** The description of GEO datasets used in this study.

Cancer type	Accession number	Total of patients	Total of methylation profiles
KIRC	GSE61441	46	229,845
BRCA	GSE69914	42	485,512
THCA	GSE86961	41	448,547
HNSC	GSE75537	29	485,512
LUSC	GSE94785	28	452,162
LIHC	GSE54503	66	485,577
COAD	GSE42752	22	485,577

*KIRC* kidney renal clear cell carcinoma, *BRCA* breast invasive carcinoma, *THCA* thyroid carcinoma, *HNSC* head and neck squamous cell carcinoma, *LUSC* lung squamous cell carcinoma, *LIHC* liver hepatocellular carcinoma, *COAD* colon adenocarcinoma.

The samples from the public databases have obtained ethical approval. And all methods were conducted in accordance with the relevant guidelines and regulations.

### Classification for cancer stage

**Figure 3:**
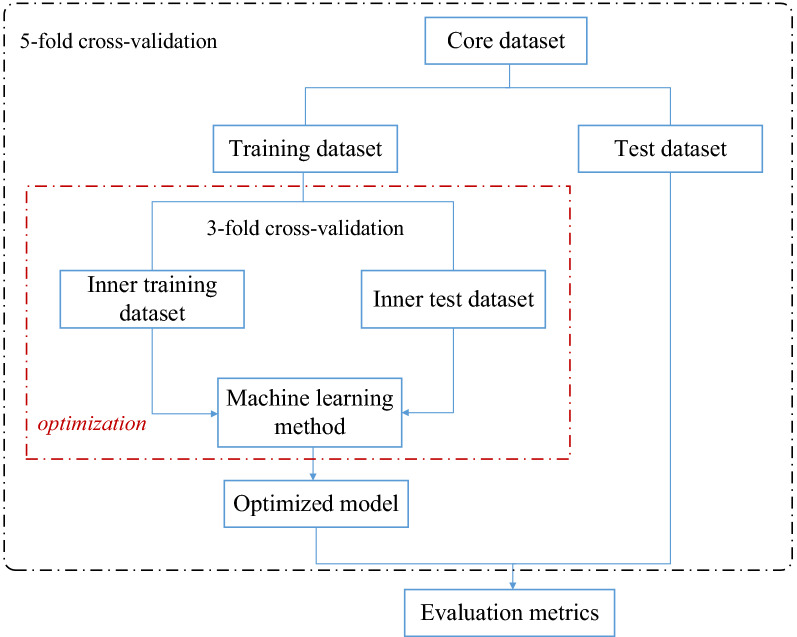
Schematic overview of the framework developed for classifying tumor stages.

We combined the samples annotated with stages I and II as early stage, and the samples annotated with stages III and IV as late stage. We used Python (version 3.7.3) and R (version 4.0.4) for data pre-processing, classification and feature selection^32^. Figure 3 describes the framework developed for cancer stage prediction.

### Extreme gradient boosting

Chen et al. developed a highly efficient and flexible gradient boosting algorithm called Extreme gradient boosting (XGBoost)^12^. It utilizes a more precise objective function and regularization term that improves its generalization ability^33^. This algorithm has been widely applied to many fields and shows its advantages in classification and regression studies^34–37^. Given a dataset $D=\left\{\left({x}_{i},{y}_{i}\right)\right\}$, here ${x}_{i}$ denotes CpG site of DNA methylation, ${y}_{i}\in \{\mathrm{0,1}\}$ is the class label of sample. Assuming that *K* is the number of trees, *F* represents the basic tree model. ${\widehat{y}}_{i}$ is denoted as the prediction of ${x}_{i}$ and given by Eq. (1):

1 $$ \hat{y}_{i} = \mathop \sum \limits_{k = 1}^{K} f_{k} \left( {x_{i} } \right), f_{k} \in F $$

where ${f}_{k}({x}_{i})$ represents the predictive score of the *k*-th tree. Equation (2) denoted the objective function of XGBoost.

2 $$ Obj = \mathop \sum \limits_{i} l\left( {\hat{y}_{i} ,y_{i} } \right) + \mathop \sum \limits_{k} {\Omega }\left( {f_{k} } \right) $$

The loss function $l$ denotes the mean square error between the prediction ${\widehat{y}}_{i}$ and the target ${y}_{i}$. The term $\Omega$ is utilized for smoothing the final learnt weights. The formula is shown as follows:

3 $$ {\Omega }\left( f \right) = \gamma T + \frac{1}{2}\lambda \left\| w \right\|^{2} $$

where $w$ denotes the score on each leaf, $T$ denotes the number of leaves, $\gamma$ and $\lambda$ represents the degrees of regularization. The objective function at the t-th iteration can be described as Eq. (4).

4 $$ Obj^{\left( t \right)} = \mathop \sum \limits_{j = 1}^{T} \left[ {\left( {\mathop \sum \limits_{{i \in I_{j} }} g_{i} } \right)w_{j} + \frac{1}{2}\left( {\mathop \sum \limits_{{i \in I_{j} }} h_{i} + \lambda } \right)w_{j}^{2} } \right] + \gamma T $$

where ${g}_{i}$ and ${h}_{i}$ refer to first and second order gradient statistics on the loss function.

### Other machine learning methods

For comprehensive analysis and comparison, we also employed other four well-known machine learning algorithms for building prediction models. Support vector machine (SVM) is a powerful supervised learning classifier^38,39^. As a kernel-based method, it aims to find optimal hyperplane that can perfectly distinguish different classes^40^. Random forest (RF) is a machine learning ensemble technique that constructs numerous decision trees based on different subsets of the data^41,42^. K-Nearest Neighbor (KNN) is a kind of simple classifier that has been extensively used for data classification^43^. Its performance is highly dependent on measuring the distance between the test samples and the training samples^44^. Naive Bayes (NB) is a probabilistic classifier that implements Bayesian techniques. The main characteristic of the classifier is that it’s robust to noise and irrelevant attributes^45–47^.

### Model optimization

In this study, we employed fivefold cross validation (fivefold CV) for assessment of the classification models. The processes were that the dataset was randomly divided into five equal folds, and taken turns to use each fold to estimate the trained model, while the remaining four folds were used to train model. The relevant parameters for each model were optimized on the training set using threefold cross validation and grid search. The classification model was trained on the training set in combination with the optimal parameters. We obtained the performance metrics of the model by averaging all results of five test sets.

For SVM, the parameters C and gamma were selected to optimize in the RBF kernel. For RF, the number of decision trees was adjusted. For KNN, we tuned hyperparameters of weights and the number of neighbors. For XGBoost, the configuration of parameters was a daunting task due to its many parameters. The optimized parameters included: ‘learning_rate’, ‘colsample_bytree’, ‘subsample’, ‘gamma’, ‘min_child_weight’, ‘max_depth’, ‘reg_lambda’, ‘reg_alpha’. Grid search made an exhaustive evaluation for various combinations of parameters and found the optimal set of parameters with the best performance.

### Performance metrics

To examine the performance of models, we utilized various evaluation metrics, commonly used to measure the classifier performances. The selected evaluation metrics include the area under the ROC curve (AUC), the area under the precision-recall curve (AUPR), accuracy (ACC), matthews correlation coefficient (MCC), Precision and Recall. FP, FN, TP and TN respectively indicate false positive, false negative, true positive and true negative predictions.

- AUC is applied to reflect the overall classification performance of the classifier by setting the discrimination threshold for comparing with the predicted probability from the classifier.
- AUPR considers the recall and precision over different thresholds.
- Accuracy is metric of model robustness and represents the percentage of correct classifications by the classifier on the test set.
  5 $$ ACC = \frac{TP + TN}{{TP + TN + FP + FN}} $$
- MCC is commonly considered as a balanced indicator that can be utilized even though the classes are heavily imbalanced.
  6 $$ MCC = \frac{TP*TN - FP*FN}{{\sqrt {TP + FP*\left( {TP + FN} \right)*\left( {TN + FP} \right)*\left( {TN + FN} \right)} }} $$
- Precision shows the ratio of correctly predicted positive samples accounts for the total number of the predicted positive samples.
  7 $$ Precision = \frac{TP}{{TP + FP}} $$
- Recall shows the ratio of correctly predicted positive samples accounts for the total number of real positive samples.
  8 $$ Recall = \frac{TP}{{TP + FN}} $$

### Feature selection with XGBoost

We utilized the XGBoost algorithm to identify the key CpG sites that differentiated early- and late-stage cancers, and these features gave insight into the biological mechanisms of cancer formation and progression. For the XGBoost algorithm, the importance score of feature can be obtained on the basis of its participation in making key decisions with boosted decision trees^12^. All input features are ranked in descending order based on their importance scores. A higher score represents that the feature is more important. We selected the top 10% CpG sites as the significant feature sets to further explore the relationship between the feature sets and cancer stage.

### Gene ontology enrichment analysis of the significant CpG sites

To explain the underlying biological mechanisms of above-mentioned important CpG sites identified by XGBoost, Gene Ontology (GO) enrichment analysis was conducted using gometh function in missMethyl package, taking into account the number of CpG sites per gene^48^. The GO terms with FDR < 0.05 were considered to be significant. Subsequently, Cytoscape (version 3.6.0) plugin ClueGO (version 2.5.7) (http://apps.cytoscape.org/apps/cluego) was utilized to cluster GO terms and showed the distribution of the clusters over the GO terms, where the kappa statistic was set to greater than or equal to 0.1^49^. By using ClueGO, the redundant GO terms were reduced and the more representative terms were preserved in our study.

### Construction of tumor specific multiclass classifier

Constructing a tumor specific classifier to identify cancer type may be valuable in the common case where the primary origin of the tumor is unknown, since determining cancer type is critical to guide more detailed diagnosis and therapy. To this aim, we built a CpG-based tumor specific model using the XGBoost algorithm that can accurately classify cancer type. We first matched nine cancer datasets (KIRC, BRCA, THCA, HNSC, KIRP, LUSC, LIHC, COAD and UCEC) from TCGA with seven corresponding cancer datasets from GEO and retained a total of 208,745 common CpG sites for every dataset. We further merged DNA methylation profiles of different cancer types from TCGA and GEO, respectively. The sample sizes of TCGA and GEO datasets are 559 and 274, respectively. XGBoost model was built using 80% of the TCGA datasets as the training set, with the remaining 20% used to assess model performance. In addition, we utilized independent GEO datasets to validate our tumor specific classifier developed using TCGA datasets, which included thirty-one CpG sites as the feature sets by XGBoost-based feature selection. For multi-classification, we used accuracy to evaluate the performance of XGBoost.

## Supplementary Information

Supplementary Information.

## Data Availability

The datasets analyzed for this study can be downloaded from The Cancer Genome Atlas (TCGA) (http://cancergenome.nih.gov/) and Gene Expression Omnibus (GEO) (https://www.ncbi.nlm.nih.gov/geo/). The code used in this study is available at https://github.com/lab319/Cancer_classification_paracancerous_tissues.
